# Predictors of linear sprint performance in professional football players

**DOI:** 10.5114/biolsport.2023.114289

**Published:** 2022-06-01

**Authors:** Joel Barrera, António J. Figueiredo, João Duarte, Adam Field, Hugo Sarmento

**Affiliations:** 1 University of Coimbra. Faculty of Sport Sciences and Physical Education, Coimbra, Portugal. Santa Clara, 3040-256 Coimbra, Portugal; 2 University of Coimbra, Research Unit for Sport and Physical Activity. Faculty of Sport Sciences and Physical Education, Coimbra, Portugal. Santa Clara, 3040-256 Coimbra, Portugal; 3 University of Huddersfield. School of Human and Health Sciences, Huddersfield, HD1 3DH, United Kingdom

**Keywords:** Strength, Speed, Evaluation, Football, Professional players

## Abstract

The purpose of this study was to analyze the relationship between sprint performance (time), and strength and power capabilities in football players. A total of 33 professional Portuguese football players performed isokinetic strength assessments, countermovement jumps (CMJ), squat jumps (SJ), and 10, 20 and 30 m sprints. Pearson’s correlation (r) was used to determine the relationships between variables. Concentric knee extensor torque at 180° · s^–1^ was largely-to-very largely correlated with 10 m (r = -0.726), 20 m (-0.657) and 30 m sprints (r = -0.823). Moderate inverse correlation were observed between CMJ (r = -0.425 and r = -0.405) and SJ height (r = -0.417 and r = -0.430), and 20 m and 30 m sprint performance, respectively. Multiple linear regression combining KEcon 180° · s^–1^ and KFcon 180° · s^–1^ demonstrated that the model was significant for predicting 10 m sprint time (F (2, 8) = 5.886; R 2 = 0.595). The model combining SJ, CMJ and KEcon 180° · s^–1^ was also significant for predicting 20 and 30 m sprint times (F (3, 7) = 2.475; R 2 = 0.515 and F (3, 7) = 5.282; R 2 = 0.562; respectively). In conclusion, peak torque at higher velocities and vertical jump performance correlates significantly with linear sprint performance (time). For practitioners seeking to improve linear sprint performance in football players, evaluation of high speed strength and vertical jump indices should be undertaken.

## INTRODUCTION

Football matches involve activity of varying speeds, with high intensity efforts performed approximately every 60 seconds [1] and accounting for ~10% of total match running. Matches elicit a greater demand on the aerobic system, although, the decisive ‘game defining’ moments are largely covered through sprint actions that require anaerobic metabolism energy delivery [1]. Previous research suggests that 83% of goals scored in the German Bundesliga were preceded by a powerful action from either the assisting or scoring player [2]. The same study also found that players completed a straight-line sprint before 45% of goals scored in the highest competitive level of German football [2]. Furthermore, coaches and sports scientists seek practical and time-efficient methods to improve top running sprint performance and acceleration capabilities in team sport athletes [3]. Accordingly, determining the important physical characteristics of professional football players will enhance understanding of the underlying determinants that influence their sprint performance.

Actions such as changes of direction, jumping, tackling, accelerating and decelerating [4], require high-force muscular contractions [1]. These activities change every 5 seconds and are carried out around 1300 times per match with ~200 of these actions performed at high or maximum intensity [5]. Therefore, the sprint performance of the movement during competitive match-play is possibly associated with explosive strength and power production, but requires further investigation. To this end, identifying the key power and strength qualities that impact linear sprint performance could help inform individualized training programs that focus on improving the key elements that influence this variable.

Explosive force plays a fundamental role in football [6], which is influenced by the interaction of different morphological and neural factors. These include muscle cross-sectional area, muscle architecture, motor unit recruitment, fibre type distribution, and neuromuscular inhibition [7]. Measurable strength and power variables, such as knee flexor and extensor muscle strength and jumping performance may predict linear sprint performance and allow differentiation between professional and amateur players [8]. Laturco et al. [9] demonstrated that vertical jump ability is significantly associated with linear and curvilinear sprint performance. A separate investigation also found that professional football players possessing higher lower extremity strength (i.e. maximum isometric knee extensor force and 1RM half squat) demonstrated superior sprint performance at 5 m and 30 m, and countermovement jump height (CMJ) [10]. Similarly, a positive relationship was found between knee extensor force (240° · s^-1^) and 30 m sprint performance in elite football players [6]. Contemporary research suggests that professional players need to significantly increase their strength to obtain slight improvements in certain running-based actions (sprint and change of direction speed) [11], controlling for strength deficits which appear to impair sprinting performance [12]. This may be due to fixture congested contemporary schedules, making it difficult to periodize strength training programmes and apply appropriate loads [13]. Therefore, due to equivocal data on predictors of linear sprint performance, more research is required.

Since the interaction between strength/power and speed is highly complex, further investigation seems warranted encompassing multiple predictor variables (e.g., jump measures and isokinetic measures) in the same multiple hierarchical linear regression models. Therefore, the objective of this study was to evaluate strength and power variables and their relationship with sprint performance (time) in professional players competing in the second highest professional division in Portugal to determine predictors of linear sprint performance.

## MATERIALS AND METHODS

### Participants

A total of 33 professional male football players from Portugal’s second highest football division (LigaPro) participated in the study (age: 26.4 ± 4.8 years, stature: 180.9 ± 6.4 cm and body mass: 77.4 ± 8.4 kg). All participants were informed of the procedures and purpose of the study and their written informed consent was obtained. The study was approved by the Ethics Committee of the University of Coimbra (CE / FCDEF-UC / 00692021) and was carried out in accordance with the Declaration of Helsinki (2013).

### Body composition

The bioelectrical impedance measurements were conducted using a valid, segmental, multi-frequency bioelectrical impedance analyzer (InBody770; Biospace, Seoul, Korea). Bioelectrical impedance has been shown to be reliable in both men and women. The intraclass correlation coefficients for body fat percentage (BF) ≥ 0.98, fat mass (FM) ≥ 0.98 and fat free mass (FFM) ≥ 0.99 and with a low standard error of measurement for BF (0.77% –0.99%), FM (0.54–0.87 kg) and FFM (0.58–0.84 kg) and a minimal difference for BF% (2.12%–2.73%), FM (1.49–2.39 kg) and FFM (1.60–2.32 kg). Participants’ age, gender and height were initially input by the lead researcher. While participants adopted a standing position with minimal clothing and barefoot, body mass assessments were performed on the scale. The participant then grasped the handles with each hand for the impedance measurements. Total body water (L), fat mass (% and kg), muscle mass (kg), and dominant lower-limb fat (kg) were measured.

### Isokinetic strength

Concentric and eccentric peak torque and mean torque (average of 5 repetitions) of the knee extensors and knee flexors were measured using an isokinetic dynamometer (Biodex Medical Systems Inc., Shirley, NY, USA). The isokinetic dynamometer is widely considered the gold standard of muscle strength testing [14] and possesses high validity and reliability [15]. Dynamometer calibration was performed as per manufacturer guidelines. A 5-min warm-up was performed, which involved participants pedaling between 50 and 60 rpm using a cycle ergometer (814E Monark, Varberg, Sweden) with a resistance corresponding to 2% body mass [16]. Participants were seated and straps were applied across the trunk, pelvis, and thigh to reduce excessive movement artefact. The crank axis of rotation was aligned to the lateral femoral epicondyle of the knee and the cuff on the padded lever arm was placed 5-cm proximal to the malleoli. Before isokinetic assessments, the tested limb remained passive and was weighed at 30° to correct for the effects of gravity [17]. The repetitions were performed through a range of 5–90° (5° close to full extension). A familiarization session was initially undertaken to minimize learning effects (three repetitions). Five repetitions were performed for each movement at angular velocities of 60 and 180° · s^-1^. Outputs were analyzed with the *Acknowledge* software version 4.1 (Biopac Systems, Inc., Goleta, CA, USA). Each curve was inspected to consider true isokinetic torques within 95% confidence limits [18].

### Countermovement Jump (CMJ) and Squat Jump (SJ)

Countermovement jump (CMJ) and squat jump (SJ) height were measured using a portable optical measurement system (Optojump, Microgate, Bolzano, Italy). The intraclass correlation coefficients (ICC) for this measurement protocol has demonstrated good reliability (ICC = 0.997–0.998). Test-retest reliability of the Optojump system has demonstrated excellent ICCs (0.982 to 0.989), low coefficients of variation (2.7% and 3.98%) and low random error (0.8 and 2.81 cm) [19, 20]. For CMJ, players were instructed to stand with their feet shoulder width apart and maintain hand-to-hip contract to negate the influence of arm swing on jump height. When prompted, participants descended rapidly into a ~60° squat position and immediately jumped vertically with maximum effort landing in the same position as takeoff. The SJ consisted of jumping from a fixed semisquat position with the hands also placed on the hips. Whilst adopting a standing position, the participants were instructed to flex at the knees to ~90° and jump maximally. The participants had to avoid as much as possible any countermovement, and they were instructed to stop for 2 seconds at each phase. Three maximal efforts were performed for both jumps with 30 seconds passive recovery between jumps; the best score was retained for analyses.

### Linear sprint performance (time) 10, 20 and 30 m shuttle

Linear sprint performance (time) was recorded for 10, 20, and 30 m sprint tests. Sprint times were recorded with three pairs of photocells (SpeedTrap II, Brower Timing Systems, Utah, USA). The photocells were placed at the start and finish (10, 20 and 30 m mark). The tests were undertaken on natural turf and conditions were standardised between repeated measures. Each sprint was performed from a standing start position and was separated by 30 seconds of active recovery. The best sprints times taken at each distance were used for analyses.

### Statistical analysis

Descriptive statistics (mean, standard error of the mean [SEM], 95% confidence interval [CI], and standard deviation [SD]) were carried out, and normality checked with the Shapiro-Wilk test. Two variables displayed non-normal distributions, and subsequently, these data were logarithmically transformed to counter the effects of non-normality. Therefore, for the first stage of data analysis, a two-tailed Pearson’s correlation (*r*) was used, with 1000 bootstrap samples. For correlations, the following criteria, whether positive or negative, were used to interpret the magnitude *of r*: < 0.1, trivial; 0.1–0.3, small; 0.3–0.5, moderate; 0.5–0.7, large; 0.7–0.9, and very large; 0.9–1.0, almost perfect [21]. All predictors that indicated a moderate (i.e., *r* = ≥ 0.3) and significant correlation were kept for further analyses, while all other variables were omitted. Multiple hierarchical linear regressions were used as appropriate with the remaining predictor variables and their dependant variable correlates. Significance and relative contribution of predictors were determined using a combination of standardized Beta values, t-statistics (i.e., the predictor makes a significant contribution to the model), and 95% confidence intervals. Statements were made regarding the magnitude of change in the dependant variables resulting from a 1 SD change (increase or decrease) in the predictor variable. All analyses were performed using the Statistical Package for the Social Sciences version 26.0 (SPSS Inc., IBM Company, Armonk, NY, USA).

## RESULTS

Table 1 presents the participants’ morphological characteristics. Descriptive statistics for sprints, jump variations and isokinetic strength are presented in Table 2.

**TABLE 1:** Descriptive statistics and normality test considering age, experience, anthropometry, and body composition for the total sample (n = 33).

variables	units	mean			SD	Shapiro-Wilk	
		value	SEM	95% CI		value	*p*
Chronological age	years	26.4	0.8	(24.7 to 28.1)	4.8	0.957	0.224
Training experience	years	17.7	1.0	(15.7 to 19.7)	5.5	0.938	0.059

Stature	cm	180.9	1.1	(178.6 to 183.2)	1.1	0.972	0.565
Body mass	kg	77.4	1.5	(74.4 to 80.4)	1.5	0.954	0.189

Total body water	L	48.4	0.8	(46.6 to 50.1)	4.9	0.985	0.925
Fat mass	%	14.2	0.5	(13.2 to 15.3)	3.0	0.978	0.751
Fat mass	kg	11.1	0.5	(10.0 to 12.2)	3.0	0.962	0.318
Muscle mass	kg	37.8	0.7	(36.4 to 39.2)	3.9	0.980	0.803
Inbody dominant lower-limb fat	kg	1.66	0.1	(1.52 to 1.81)	0.4	0.945	0.098

Abbreviations: SEM (standard error of the mean); CI (confidence interval); SD (standard deviation).

**TABLE 2:** Descriptive statistics and normality test considering acceleration, speed/velocity, strength and jumping performance for the total sample (n = 33).

variables	units	mean			SD	Shapiro-Wilk	
		value	SEM	95% CI		value	*p*
Sprint 10 m	s	1.57	0.02	(1.52 to 1.61)	0.13	0.952	0.163
Sprint 20 m	s	2.99	0.03	(2.92 to 3.05)	0.03	0.937	0.063
Sprint 30 m	s	4.55	0.04	(4.46 to 4.63)	0.24	0.973	0.589

Squat jump (SJ)	cm	36.0	0.8	(34.4 to 37.6)	4.4	0.975	0.660
Countermovement jump (CMJ)	cm	37.2	1.0	(35.2 to 39.1)	5.5	0.969	0.483

KE isokinetic eccentric muscular action 60°/s	Nm	254.1	7.4	(238.9 to 269.2)	42.7	0.963	0.321
KF isokinetic concentric muscular action 60°/s	Nm	146.0	4.0	(137.8 to 154.2)	23.1	0.964	0.352
KE isokinetic eccentric muscular action 60°/s	Nm	317.0	13.4	(289.8 to 344.2)	76.7	0.983	0.887
KF isokinetic concentric muscular action 60°/s	Nm	197.4	6.5	(184.1 to 210.8)	37.6	0.957	0.224
KE isokinetic eccentric muscular action 180°/s	Nm	170.5	6.6	(155.6 to 185.3)	22.1	0.967	0.853
KF isokinetic concentric muscular action 180°/s	Nm	110.1	4.0	(101.1 to 119.1)	13.4	0.920	0.315
KE isokinetic eccentric muscular action 180°/s	Nm	285.9	15.2	(257.5 to 312.9)	45.6	0.921	0.404
KF isokinetic concentric muscular action 180°/s	Nm	170.5	7.6	(154.9 to 185.0)	22.7	0.972	0.915

Abbreviations: SEM (standard error of the mean); CI (confidence interval); SD (standard deviation).

Moderate and significant correlations are outlined in Table 3 for four predictor variables (SJ, CMJ height, KEcon and KFcon). The multiple regression model combining KE and KF at 180º/s was found to be significant for predicting 10 m (F (2, 8) = 5.886: p < 0.05; R 2 = 0.595), 20 m (F (3, 7) = 2.475: p = 0.146; R 2 = 0.515) and 30 m sprint times (F (3, 7) = 5.282: p < 0.05; R 2 = 0.562) (Table 4).

**TABLE 3:** Bivariate correlations between strength and sprint performance.

Variable	units	Linear sprinting (s)					
		10 m		20 m		30 m	
		*p*	*r*	*p*	*r*	*p*	*r*
Squat jump (SJ) height	cm	0.198	–0.230	0.018	–0.417*	0.014	–0.430*
Countermovement jump (CMJ) height	cm	0.347	–0.169	0.015	–0.425*	0.022	–0.405*

KE isokinetic concentric muscular action 60°/s	Nm	0.895	0.024	0.089	–0.306	0.273	–0.200
KF isokinetic concentric muscular action 60°/s	Nm	0.230	0.215	0.203	–0.231	0.836	–0.038
KE isokinetic eccentric muscular action 60°/s	Nm	0.927	–0.017	0.388	–0.158	0.698	–0.071
KF isokinetic eccentric muscular action 60°/s	Nm	0.735	–0.061	0.472	–0.132	0.682	–0.075
KE isokinetic concentric muscular action 180°/s	Nm	0.011	–0.726*	0.028	–0.657*	0.002	–0.823**
KF isokinetic concentric muscular action 180°/s	Nm	0.014	–0.712*	0.387	–0.290	0.080	–0.549
KE isokinetic eccentric muscular action 180°/s	Nm	0.551	–0.230	0.186	–0.485	0.188	–0.483
KF isokinetic eccentric muscular action 180°/s	Nm	0.109	0.570	0.705	0.148	0.653	–0.175

Abbreviations: BD (bilateral difference); PT (peak torque); T (torque); KE (knee extension); KF (knee flexion).

* p < 0.05;

** p < 0.001.

**TABLE 4:** Unstandardized and standardized Beta values for each regression models.

Assessment	Variable	*B*	SE	*ß*
10 m sprint	Constant	2.476	0.261	
	KE isokinetic concentric muscular action 180°/s	−0.003	0.002	−0.440
	KF isokinetic concentric muscular action 180°/s	−0.004	0.003	−0.388

20 m sprint	Constant	4.106	0.521	
	Squat jump (SJ) height	0.019	0.033	0.411
	Countermovement jump (CMJ) height	−0.030	0.032	−0.710
	KE isokinetic concentric muscular action 180°/s	−0.004	0.003	−0.428

30 m sprint	Constant	6.633	0.566	
	Squat jump (SJ) height	−0.021	0.036	−0.330
	Countermovement jump (CMJ) height	0.018	0.035	0.312
	KE isokinetic concentric muscular action 180°/s	−0.011	0.003	−0.858*

* Standardized beta values are significant at the P < 0.05 level.

## DISCUSSION

Based on the notion that linear sprint time is key for success in professional football [2], the main objective of this study was to determine the relationship between linear sprint time and other physical performance predictors in elite football players. Large and very large relationships were found between 10 m sprint time and KEcon and KFcon at 180° · s^–1^, 20 m sprint time and SJ, CMJ and KEcon at 180° · s^–1^, and 30 m sprint time and SJ, CMJ and KEcon at 180° · s^–1^. Multiple regression analysis evaluates the strength of the relationship between the dependent variable and several predictor variables to facilitate robust conclusions. Such data can be utilised by strength and physical conditioning practitioners to adapt training protocols and optomize linear sprint performance in professional football players.

The current findings that knee extensor strength at higher velocities was correlated with sprint speed are consistent with data derived from similar research [6]. This investigation demonstrated that there was a significant relationship between knee extensor peak torque at 240° · s^-1^ and 30 m sprint performance. Knee extensor peak torque is thought to be a key determinant for performing rapid knee extension during the swing phase while sprinting [22]. Sprint performance has also shown to better correlated with knee extensor peak torque at 240° · s^-1^ than the knee flexors [23]. Furthermore, a separate investigation reported stronger correlations with isokinetic knee extension strength with faster velocities (150 and 240° · s^-1^) versus slower speeds of 60° · s^-1^ [24]. Therefore, it seems that the ability to produce greater knee extensor peak torque at high velocities is a positive factor for sprint performance. As such, knee extensor strength may need to be developed at higher speeds in order for sprint ability to be improved. Future research should assess whether training interventions targetting high velocity knee extensor peak torque translates to improvements in sprint performance.

The SJ and CMJ jump tests showed a moderate inverse correlation with 20 and 30 m sprint performance, but no associations with 10 m sprint performance were identified. These findings are similar to those published in previous studies, suggesting that relative peak power output during the bilateral CMJ is negatively associated with linear sprinting performance over 5, 10, and 20 m in English Premier League football players [25]. The findings are also somewhat analogous with another study evaluating 25 professional football players, with strong and negative correlations between CMJ and 30 m sprint performance, but no relationships with 10 m sprint performance [10]. Therefore, vertical jump height appears to be negatively correlated with sprint performance only at higher distances (i.e., 20 and 30 m). This notion may be related to how the transition from lower to higher speeds results in shorter stance phase duration, with a concomitant increase in maximum vertical force production [26, 27]. This inturn leads to an increase in flight time and stride length [28], but these factors may not have chance to influence sprint time over such short distances [26], perhaps explaining the lack of correlation for 10 m sprint performance. The ability to produce maximum force rapidly is a requirement for explosive jump performance – which is also a prerequisite for rapidly accelerating over short distances and reaching high speeds [10]. These actions are intergral moments in match-play and occur during the decisive phases of play [29]. Therefore, this information is important for practitioners with a training focus geared towards developing linear sprint and acceleration capacity.

The current study provides novel insights into the physical determinants of linear sprint performance in football although, the findings must be considered within the constraints of professional sports. Firstly, there are an absence of measures assessing horizontal jump performance, despite previous research demonstrating that horizontal ground reaction forces are more predominantly involved in top-speed acceleration [30]. Therefore, this must be considered a limitation since horizontal jumping and the associated force vectors may be better predictors of linear sprint performance than solely vertical jump performance and isokinetic strength. Additionally, football players rarely accelerate from a stationary position to maximum speed during matches [31] but are more likely to increase speeds from low-velocity movements. Therefore, the ecological validity of the sprinting mechanics with reference to their translational application in football may be questionable. Further studies should assess the predictive ability of horizontal ground forces across different positional roles in football.

## CONCLUSIONS

The results in the present study suggest that strength and power variables at faster speeds are associated with linear sprint performance. Professional football players with greater torque at 180° · s^-1^ had faster sprint times at 10, 20 and 30 m. It is recommended that staff responsible for implementing training regimes should design training protocols that develop knee extensor strength at a higher rate to optimize running performance in professional football players. Another key finding demonstrated that SJ and CMJ are negatively correlated with 20 and 30 m sprint performance. Such relationships between vertical force generation and sprint times indicate that training protocols aimed at increasing lower extremity explosive strength should be incorporated into weekly football microcycles to maximize sprint performance. Future research avenues should explore the predictive capacity of reactive strength markers and horizontal jump indices in football players of different playing levels.
